# 2-(4-Methyl­cyclo­hex-3-en­yl)propan-2-yl *N*-phenyl­carbamate

**DOI:** 10.1107/S1600536810025080

**Published:** 2010-07-03

**Authors:** Raza Murad Ghalib, Othman Sulaiman, Sayed Hasan Mehdi, Jia Hao Goh, Hoong-Kun Fun

**Affiliations:** a School of Industrial Technology, Universiti Sains Malaysia, 11800 USM, Penang, Malaysia; b X-ray Crystallography Unit, School of Physics, Universiti Sains Malaysia, 11800 USM, Penang, Malaysia

## Abstract

In the title carbamate compound, C_17_H_23_NO_2_, one of the C*sp* ^3^ atoms of the cyclo­hexene ring is disordered over two sites with refined occupancies of 0.55 (2) and 0.45 (2), both disorder components resulting in half-boat conformations. The mean plane through the carbamate unit is inclined at inter­planar angles of 14.80 (13), 18.30 (17) and 24.0 (2)°, respectively, with respect to the phenyl ring, and the major and minor disorder component cyclo­hexene rings. In the crystal structure, adjacent mol­ecules are linked into chains along [001] *via* inter­molecular N—H⋯O hydrogen bonds. The crystal structure is further stabilized by weak inter­molecular C—H⋯π inter­actions.

## Related literature

For general background to and applications of the title compound, see: Banerjee *et al.* (1978 ▶); Graia *et al.* (2009 ▶); Ibuka *et al.* (1985 ▶); Lapidus *et al.* (1987 ▶); Loev & Kormendy (1963 ▶); Muradov *et al.* (1986 ▶); Niu *et al.* (2007 ▶); Ibuka *et al.* (1985 ▶). For related carbamate structures, see: Garden *et al.* (2007 ▶); Graia *et al.* (2009 ▶). For the stability of the temperature controller used for the data collection, see: Cosier & Glazer (1986 ▶).
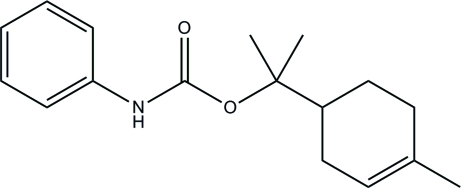


## Experimental

### 

#### Crystal data

C_17_H_23_NO_2_
*M* *_r_* = 273.36
Monoclinic, 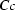

*a* = 19.3067 (19) Å
*b* = 9.0058 (9) Å
*c* = 8.9521 (9) Å
β = 100.964 (3)°
*V* = 1528.1 (3) Å^3^
*Z* = 4
Mo *K*α radiation
μ = 0.08 mm^−1^
*T* = 100 K
0.58 × 0.20 × 0.10 mm

#### Data collection

Bruker APEXII DUO CCD diffractometer
Absorption correction: multi-scan (*SADABS*; Bruker, 2009 ▶) *T*_min_ = 0.957, *T*_max_ = 0.992
8744 measured reflections
2205 independent reflections
2053 reflections with *I* > 2σ(*I*)
*R*_int_ = 0.049

#### Refinement

*R*[*F* ^2^ > 2σ(*F* ^2^)] = 0.050
*wR*(*F* ^2^) = 0.154
*S* = 1.15
2205 reflections
187 parameters
4 restraints
H-atom parameters constrained
Δρ_max_ = 0.68 e Å^−3^
Δρ_min_ = −0.76 e Å^−3^

### 

Data collection: *APEX2* (Bruker, 2009 ▶); cell refinement: *SAINT* (Bruker, 2009 ▶); data reduction: *SAINT*; program(s) used to solve structure: *SHELXTL* (Sheldrick, 2008 ▶); program(s) used to refine structure: *SHELXTL*; molecular graphics: *SHELXTL*; software used to prepare material for publication: *SHELXTL* and *PLATON* (Spek, 2009 ▶).

## Supplementary Material

Crystal structure: contains datablocks global, I. DOI: 10.1107/S1600536810025080/hb5524sup1.cif

Structure factors: contains datablocks I. DOI: 10.1107/S1600536810025080/hb5524Isup2.hkl

Additional supplementary materials:  crystallographic information; 3D view; checkCIF report

## Figures and Tables

**Table 1:** Hydrogen-bond geometry (Å, °) *Cg*1 is the centroid of the C1–C6 phenyl ring.

*D*—H⋯*A*	*D*—H	H⋯*A*	*D*⋯*A*	*D*—H⋯*A*
N1—H1*N*1⋯O2^i^	0.86	2.12	2.969 (3)	170
C13—H13*A*⋯*Cg*1^ii^	0.97	2.62	3.566 (3)	166

Symmetry codes: (i) 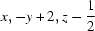; (ii) 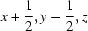.

**Fig. 1.**
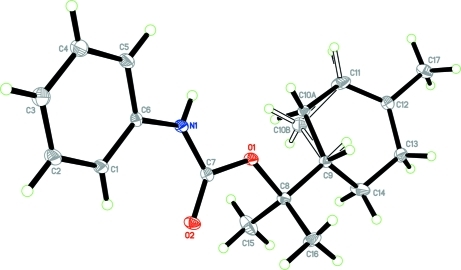
The molecular structure of (I), showing 30 % probability displacement ellipsoids. Open bonds indicate the minor disordered component.

**Fig. 2.**
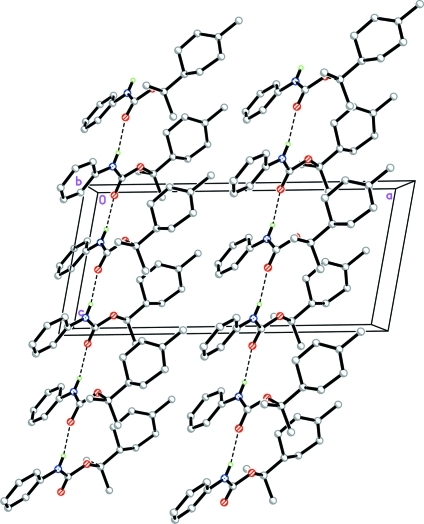
The crystal structure of (I), viewed down the b axis, showing molecules being linked into one-dimensional chains along the c axis. Minor disordered component and H atoms not involved in intermolecular hydrogen bonds (dashed lines) have been omitted for clarity.

## supplementary crystallographic information

### Comment

Carbamates are well-known class of compounds with biological activity (Muradov *et al.*, 1986). They can be prepared by different methods, for example by nickel-catalyzed coupling of CO_2_ and amines (Niu *et al.*, 2007), by stirring of alcohols including steroids as well as primary and secondary alcohols, polyols, phenols with sodium cynate, and trifluoroacetic acid (Loev & Kormendy, 1963), by carbonylation of aromatic nitro compounds (Lapidus *et al.*, 1987), by the reaction of isocynates with alcohols (Ibuka *et al.*, 1985) in the presence of lewis acid and by the reaction of an amine and an alcohol with phosgene. Phytosterol, β-Sitosterol, stigmasterol and cholesterol react with phenyl isocyanate to give carbamate (Banerjee *et al.*, 1978; Graia *et al.*, 2009). In this study the title compound has been synthesized by the reaction of α-terpineol with phenylisocyanate in the presence of catalytic amount of HCl in chloroform solvent.

In the title carbamate compound (Fig. 1), atom C10 of the cyclohexene ring (C9-C14) is disordered over two sites with a refined occupancy ratio of 0.55 (2):0.45 (2). The major (C9/C10A/C11-C14) and minor (C9/C10B/C11-C14) disordered cyclohexene rings adopt the same conformation, that is the half-boat conformation; puckering parameters Q = 0.427 (4) Å, θ = 57.4 (5)°, φ = 335.9 (7)° for major disordered component and Q = 0.651 (6) Å, θ = 131.6 (4)° and φ = 161.7 (7)° for minor disordered component. The mean plane through the carbamate moiety (N1/C7/O1/O2) is inclined at interplanar angles of 14.80 (13), 18.30 (17) and 24.0 (2)°, respectively, with respect to the C1-C6 phenyl ring, major and minor disordered cyclohexene rings. The bond lengths and angles are comparable to those related carbamate structures (Garden *et al.*, 2007; Graia *et al.*, 2009).

In the crystal structure, intermolecular N1—H1N1···O2 hydrogen bonds (Table 1) link adjacent molecules into one-dimensional chains running along the [001] direction (Fig. 2). Further stabilization of the crystal structure is provided by weak intermolecular C13—H13A···*Cg*1 interactions (Table 1) involving the centroid of the C1-C6 phenyl ring.

### Experimental

A mixture of α-terpineol (1.640 ml) and phenylisocyanate (1.087 ml) in 1:1 molar ratio were stirred in chloroform for 30 minutes in the presence of catalytic amount of HCl. The reaction mixture was dried on rota vapor at low pressure and then chromatographed over silica gel column loaded in light petroleum ether. The column was eluted only with light petroleum ether to give five fractions of the title compound. These fractions were mixed together on the basis of same TLC results and crystallized with chloroform:alcohol (1:1) to give the colourless needles of (I) (1.93 g, *M.p.* 378 K). The melting point was taken on Thermo Fisher digital melting point apparatus of IA9000 series and is uncorrected. Open column chromatography was performed on silica gel 60 (Merck, 0.040–0.063 mm, 230–400 mesh ASTM) and Sephadex LH-20 (Pharmacia). TLCs were taken on silica gel plates (silica gel 60 F_254_ on aluminum foil, Merck).

### Refinement

Atom C10 is disordered over two sites with a refined occupancy ratio of 0.55 (2):0.45 (2). Atom C10B of the minor disordered component was refined isotropically. The C—C bond lengths in the minor disordered component were restrained with distance of 1.50 (1) Å. All H atoms were placed in their calculated positions, with N—H = 0.86 and C—H = 0.93 or 0.96 Å, and refined using a riding model, with *U*_iso_ = 1.2 *U*_eq_(N) and *U*_iso_ = 1.2 or 1.5 *U*_eq_(C). The rotating group model was applied to the methyl groups. In the absence of significant anomalous dispersion, 1491 Friedel pairs were merged in the final refinement.

### Crystal data

**Table:**

C_17_H_23_NO_2_	*F*(000) = 592
*M**_r_* = 273.36	*D*_x_ = 1.188 Mg m^−^^3^
Monoclinic, *C**c*	Mo *K*α radiation, λ = 0.71073 Å
Hall symbol: C -2yc	Cell parameters from 2414 reflections
*a* = 19.3067 (19) Å	θ = 3.3–32.4°
*b* = 9.0058 (9) Å	µ = 0.08 mm^−^^1^
*c* = 8.9521 (9) Å	*T* = 100 K
β = 100.964 (3)°	Needle, colourless
*V* = 1528.1 (3) Å^3^	0.58 × 0.20 × 0.10 mm
*Z* = 4	

### Data collection

**Table:**

Bruker APEXII DUO CCD diffractometer	2205 independent reflections
Radiation source: fine-focus sealed tube	2053 reflections with *I* > 2σ(*I*)
graphite	*R*_int_ = 0.049
φ and ω scans	θ_max_ = 30.0°, θ_min_ = 2.5°
Absorption correction: multi-scan (*SADABS*; Bruker, 2009)	*h* = −26→27
*T*_min_ = 0.957, *T*_max_ = 0.992	*k* = −12→11
8744 measured reflections	*l* = −12→12

### Refinement

**Table:**

Refinement on *F*^2^	Secondary atom site location: difference Fourier map
Least-squares matrix: full	Hydrogen site location: inferred from neighbouring sites
*R*[*F*^2^ > 2σ(*F*^2^)] = 0.050	H-atom parameters constrained
*wR*(*F*^2^) = 0.154	*w* = 1/[σ^2^(*F*_o_^2^) + (0.0981*P*)^2^ + 0.223*P*] where *P* = (*F*_o_^2^ + 2*F*_c_^2^)/3
*S* = 1.15	(Δ/σ)_max_ < 0.001
2205 reflections	Δρ_max_ = 0.68 e Å^−^^3^
187 parameters	Δρ_min_ = −0.76 e Å^−^^3^
4 restraints	Extinction correction: *SHELXTL* (Sheldrick, 2008), Fc^*^=kFc[1+0.001xFc^2^λ^3^/sin(2θ)]^-1/4^
Primary atom site location: structure-invariant direct methods	Extinction coefficient: 0.044 (6)

### Special details

**Table:**

Experimental. The crystal was placed in the cold stream of an Oxford Cryosystems Cobra open-flow nitrogen cryostat (Cosier & Glazer, 1986) operating at 100.0 (1)K.
Geometry. All esds (except the esd in the dihedral angle between two l.s. planes) are estimated using the full covariance matrix. The cell esds are taken into account individually in the estimation of esds in distances, angles and torsion angles; correlations between esds in cell parameters are only used when they are defined by crystal symmetry. An approximate (isotropic) treatment of cell esds is used for estimating esds involving l.s. planes.
Refinement. Refinement of F^2^ against ALL reflections. The weighted R-factor wR and goodness of fit S are based on F^2^, conventional R-factors R are based on F, with F set to zero for negative F^2^. The threshold expression of F^2^ > 2sigma(F^2^) is used only for calculating R-factors(gt) etc. and is not relevant to the choice of reflections for refinement. R-factors based on F^2^ are statistically about twice as large as those based on F, and R- factors based on ALL data will be even larger.

### Fractional atomic coordinates and isotropic or equivalent isotropic displacement parameters (Å^2^)

**Table:**

	*x*	*y*	*z*	*U*_iso_*/*U*_eq_	Occ. (<1)
O1	0.15396 (9)	0.88990 (19)	0.87624 (19)	0.0181 (4)	
O2	0.08897 (11)	0.8780 (2)	1.0641 (2)	0.0208 (4)	
N1	0.07470 (11)	1.0610 (2)	0.8828 (2)	0.0164 (4)	
H1N1	0.0841	1.0796	0.7946	0.020*	
C1	−0.00477 (14)	1.1337 (3)	1.0559 (3)	0.0212 (5)	
H1A	−0.0006	1.0410	1.1025	0.025*	
C2	−0.04625 (16)	1.2429 (3)	1.1041 (3)	0.0272 (6)	
H2A	−0.0697	1.2221	1.1834	0.033*	
C3	−0.05361 (16)	1.3820 (3)	1.0371 (4)	0.0286 (6)	
H3A	−0.0810	1.4547	1.0716	0.034*	
C4	−0.01903 (15)	1.4106 (3)	0.9166 (3)	0.0264 (6)	
H4A	−0.0244	1.5025	0.8684	0.032*	
C5	0.02310 (14)	1.3035 (3)	0.8685 (3)	0.0224 (5)	
H5A	0.0467	1.3246	0.7897	0.027*	
C6	0.03042 (11)	1.1636 (3)	0.9376 (3)	0.0155 (4)	
C7	0.10429 (12)	0.9363 (3)	0.9525 (3)	0.0165 (4)	
C8	0.19360 (13)	0.7514 (3)	0.9145 (3)	0.0180 (5)	
C9	0.24506 (13)	0.7554 (3)	0.8015 (3)	0.0161 (4)	
H9A	0.2783	0.8351	0.8398	0.019*	0.55 (2)
H9B	0.2736	0.8434	0.8163	0.019*	0.45 (2)
C10A	0.2126 (3)	0.8036 (10)	0.6393 (5)	0.0169 (17)	0.55 (2)
H10A	0.1711	0.7435	0.6029	0.020*	0.55 (2)
H10B	0.1974	0.9062	0.6412	0.020*	0.55 (2)
C10B	0.2058 (3)	0.7435 (16)	0.6365 (7)	0.025 (2)*	0.45 (2)
H10C	0.1653	0.8094	0.6189	0.030*	0.45 (2)
H10D	0.1895	0.6426	0.6136	0.030*	0.45 (2)
C11	0.26080 (18)	0.7903 (4)	0.5329 (3)	0.0338 (7)	
H11A	0.2476	0.8317	0.4365	0.041*	0.55 (2)
H11B	0.2506	0.8616	0.4521	0.041*	0.45 (2)
C12	0.32574 (14)	0.7183 (3)	0.5690 (3)	0.0205 (5)	
C13	0.34562 (13)	0.6355 (3)	0.7083 (3)	0.0227 (5)	
H13A	0.3867	0.6828	0.7687	0.027*	
H13B	0.3598	0.5369	0.6825	0.027*	
C14	0.2908 (2)	0.6188 (4)	0.8067 (4)	0.0392 (9)	
H14A	0.3141	0.6005	0.9110	0.047*	
H14B	0.2612	0.5336	0.7724	0.047*	
C15	0.14268 (18)	0.6215 (3)	0.8893 (5)	0.0355 (7)	
H15A	0.1125	0.6249	0.9629	0.053*	
H15B	0.1688	0.5301	0.9002	0.053*	
H15C	0.1146	0.6272	0.7887	0.053*	
C16	0.23442 (17)	0.7591 (4)	1.0772 (3)	0.0316 (7)	
H16A	0.2020	0.7563	1.1463	0.047*	
H16B	0.2610	0.8497	1.0915	0.047*	
H16C	0.2660	0.6760	1.0966	0.047*	
C17	0.37752 (17)	0.7262 (3)	0.4634 (3)	0.0271 (5)	
H17A	0.3578	0.7843	0.3757	0.041*	
H17B	0.3874	0.6278	0.4321	0.041*	
H17C	0.4204	0.7716	0.5151	0.041*	

### Atomic displacement parameters (Å^2^)

**Table:**

	*U*^11^	*U*^22^	*U*^33^	*U*^12^	*U*^13^	*U*^23^
O1	0.0224 (8)	0.0176 (8)	0.0163 (8)	0.0042 (6)	0.0091 (6)	0.0011 (6)
O2	0.0282 (9)	0.0201 (9)	0.0167 (8)	0.0026 (7)	0.0113 (7)	0.0013 (6)
N1	0.0198 (9)	0.0193 (10)	0.0116 (8)	0.0022 (7)	0.0069 (7)	−0.0003 (7)
C1	0.0232 (11)	0.0244 (13)	0.0184 (11)	0.0048 (9)	0.0095 (9)	0.0040 (9)
C2	0.0319 (14)	0.0304 (15)	0.0241 (12)	0.0080 (10)	0.0172 (11)	0.0051 (10)
C3	0.0343 (14)	0.0255 (14)	0.0302 (14)	0.0102 (11)	0.0166 (12)	0.0004 (10)
C4	0.0309 (13)	0.0208 (12)	0.0306 (14)	0.0034 (10)	0.0137 (11)	0.0018 (10)
C5	0.0237 (11)	0.0207 (13)	0.0258 (12)	0.0019 (9)	0.0126 (9)	0.0028 (9)
C6	0.0141 (9)	0.0177 (11)	0.0152 (9)	−0.0004 (8)	0.0042 (7)	−0.0019 (8)
C7	0.0176 (10)	0.0186 (11)	0.0140 (10)	−0.0009 (8)	0.0051 (8)	−0.0041 (8)
C8	0.0220 (11)	0.0145 (11)	0.0191 (11)	0.0041 (8)	0.0081 (8)	0.0015 (8)
C9	0.0184 (9)	0.0163 (11)	0.0147 (10)	0.0013 (8)	0.0057 (8)	−0.0006 (8)
C10A	0.020 (2)	0.018 (4)	0.014 (2)	0.0052 (19)	0.0056 (14)	0.0028 (15)
C11	0.0466 (17)	0.0417 (17)	0.0160 (12)	0.0201 (14)	0.0135 (12)	0.0090 (11)
C12	0.0223 (11)	0.0227 (12)	0.0180 (10)	0.0001 (9)	0.0079 (9)	−0.0042 (9)
C13	0.0210 (11)	0.0260 (13)	0.0227 (12)	0.0058 (9)	0.0079 (9)	−0.0001 (9)
C14	0.0482 (17)	0.0328 (16)	0.0466 (19)	0.0244 (14)	0.0343 (16)	0.0214 (14)
C15	0.0387 (15)	0.0185 (13)	0.057 (2)	−0.0059 (11)	0.0284 (15)	−0.0076 (12)
C16	0.0340 (14)	0.0489 (18)	0.0134 (11)	0.0186 (13)	0.0082 (10)	0.0086 (11)
C17	0.0346 (13)	0.0235 (13)	0.0273 (13)	−0.0006 (10)	0.0163 (11)	−0.0031 (10)

### Geometric parameters (Å, °)

**Table:**

O1—C7	1.345 (3)	C10A—C11	1.458 (5)
O1—C8	1.470 (3)	C10A—H10A	0.9700
O2—C7	1.214 (3)	C10A—H10B	0.9700
N1—C7	1.356 (3)	C10B—C11	1.594 (7)
N1—C6	1.409 (3)	C10B—H10C	0.9700
N1—H1N1	0.8600	C10B—H10D	0.9700
C1—C2	1.388 (4)	C11—C12	1.393 (4)
C1—C6	1.389 (3)	C11—H11A	0.9300
C1—H1A	0.9300	C11—H11B	0.9600
C2—C3	1.385 (4)	C12—C13	1.441 (4)
C2—H2A	0.9300	C12—C17	1.502 (3)
C3—C4	1.396 (4)	C13—C14	1.509 (4)
C3—H3A	0.9300	C13—H13A	0.9700
C4—C5	1.383 (4)	C13—H13B	0.9700
C4—H4A	0.9300	C14—H14A	0.9700
C5—C6	1.399 (4)	C14—H14B	0.9700
C5—H5A	0.9300	C15—H15A	0.9600
C8—C15	1.517 (4)	C15—H15B	0.9600
C8—C16	1.520 (4)	C15—H15C	0.9600
C8—C9	1.547 (3)	C16—H16A	0.9600
C9—C14	1.510 (4)	C16—H16B	0.9600
C9—C10B	1.531 (6)	C16—H16C	0.9600
C9—C10A	1.531 (5)	C17—H17A	0.9600
C9—H9A	0.9800	C17—H17B	0.9600
C9—H9B	0.9600	C17—H17C	0.9600
			
C7—O1—C8	122.38 (19)	H10A—C10A—H10B	107.7
C7—N1—C6	127.81 (19)	C9—C10B—C11	106.2 (5)
C7—N1—H1N1	116.1	C9—C10B—H10C	110.5
C6—N1—H1N1	116.1	C11—C10B—H10C	110.5
C2—C1—C6	119.6 (2)	C9—C10B—H10D	110.5
C2—C1—H1A	120.2	C11—C10B—H10D	110.5
C6—C1—H1A	120.2	H10C—C10B—H10D	108.7
C3—C2—C1	121.6 (3)	C12—C11—C10A	123.1 (3)
C3—C2—H2A	119.2	C12—C11—C10B	114.4 (4)
C1—C2—H2A	119.2	C12—C11—H11A	118.5
C2—C3—C4	118.5 (3)	C10A—C11—H11A	118.5
C2—C3—H3A	120.8	C10B—C11—H11A	123.4
C4—C3—H3A	120.8	C12—C11—H11B	122.2
C5—C4—C3	120.5 (3)	C10A—C11—H11B	111.8
C5—C4—H4A	119.7	C10B—C11—H11B	123.4
C3—C4—H4A	119.7	C11—C12—C13	121.4 (2)
C4—C5—C6	120.4 (2)	C11—C12—C17	120.6 (2)
C4—C5—H5A	119.8	C13—C12—C17	118.0 (2)
C6—C5—H5A	119.8	C12—C13—C14	117.1 (2)
C1—C6—C5	119.3 (2)	C12—C13—H13A	108.0
C1—C6—N1	123.8 (2)	C14—C13—H13A	108.0
C5—C6—N1	117.0 (2)	C12—C13—H13B	108.0
O2—C7—O1	126.3 (2)	C14—C13—H13B	108.0
O2—C7—N1	126.1 (2)	H13A—C13—H13B	107.3
O1—C7—N1	107.62 (19)	C13—C14—C9	111.8 (2)
O1—C8—C15	109.0 (2)	C13—C14—H14A	109.3
O1—C8—C16	109.7 (2)	C9—C14—H14A	109.3
C15—C8—C16	112.4 (3)	C13—C14—H14B	109.3
O1—C8—C9	101.42 (18)	C9—C14—H14B	109.3
C15—C8—C9	113.5 (2)	H14A—C14—H14B	107.9
C16—C8—C9	110.2 (2)	C8—C15—H15A	109.5
C14—C9—C10B	98.7 (5)	C8—C15—H15B	109.5
C14—C9—C10A	113.1 (3)	H15A—C15—H15B	109.5
C14—C9—C8	113.9 (2)	C8—C15—H15C	109.5
C10B—C9—C8	111.6 (3)	H15A—C15—H15C	109.5
C10A—C9—C8	115.4 (2)	H15B—C15—H15C	109.5
C14—C9—H9A	104.3	C8—C16—H16A	109.5
C10B—C9—H9A	124.1	C8—C16—H16B	109.5
C10A—C9—H9A	104.3	H16A—C16—H16B	109.5
C8—C9—H9A	104.3	C8—C16—H16C	109.5
C14—C9—H9B	110.6	H16A—C16—H16C	109.5
C10B—C9—H9B	111.0	H16B—C16—H16C	109.5
C10A—C9—H9B	91.0	C12—C17—H17A	109.5
C8—C9—H9B	110.7	C12—C17—H17B	109.5
C11—C10A—C9	113.5 (3)	H17A—C17—H17B	109.5
C11—C10A—H10A	108.9	C12—C17—H17C	109.5
C9—C10A—H10A	108.9	H17A—C17—H17C	109.5
C11—C10A—H10B	108.9	H17B—C17—H17C	109.5
C9—C10A—H10B	108.9		
			
C6—C1—C2—C3	0.0 (5)	O1—C8—C9—C10A	−43.0 (5)
C1—C2—C3—C4	1.0 (5)	C15—C8—C9—C10A	73.8 (5)
C2—C3—C4—C5	−1.7 (5)	C16—C8—C9—C10A	−159.1 (4)
C3—C4—C5—C6	1.4 (5)	C14—C9—C10A—C11	−40.1 (8)
C2—C1—C6—C5	−0.3 (4)	C10B—C9—C10A—C11	−89.5 (9)
C2—C1—C6—N1	178.8 (2)	C8—C9—C10A—C11	−173.6 (5)
C4—C5—C6—C1	−0.4 (4)	C14—C9—C10B—C11	−74.1 (7)
C4—C5—C6—N1	−179.5 (2)	C10A—C9—C10B—C11	60.9 (8)
C7—N1—C6—C1	−17.3 (4)	C8—C9—C10B—C11	165.8 (5)
C7—N1—C6—C5	161.8 (2)	C9—C10A—C11—C12	9.9 (9)
C8—O1—C7—O2	4.6 (4)	C9—C10A—C11—C10B	80.2 (9)
C8—O1—C7—N1	−175.8 (2)	C9—C10B—C11—C12	49.7 (9)
C6—N1—C7—O2	12.6 (4)	C9—C10B—C11—C10A	−70.3 (9)
C6—N1—C7—O1	−166.9 (2)	C10A—C11—C12—C13	8.1 (7)
C7—O1—C8—C15	62.9 (3)	C10B—C11—C12—C13	−13.0 (6)
C7—O1—C8—C16	−60.6 (3)	C10A—C11—C12—C17	−171.4 (5)
C7—O1—C8—C9	−177.0 (2)	C10B—C11—C12—C17	167.5 (5)
O1—C8—C9—C14	−176.2 (3)	C11—C12—C13—C14	5.2 (4)
C15—C8—C9—C14	−59.4 (3)	C17—C12—C13—C14	−175.3 (3)
C16—C8—C9—C14	67.7 (3)	C12—C13—C14—C9	−35.1 (4)
O1—C8—C9—C10B	−65.4 (6)	C10B—C9—C14—C13	68.3 (5)
C15—C8—C9—C10B	51.3 (6)	C10A—C9—C14—C13	52.4 (5)
C16—C8—C9—C10B	178.4 (6)	C8—C9—C14—C13	−173.4 (3)

### Hydrogen-bond geometry (Å, °)

**Table:**

Cg1 is the centroid of C1–C6 phenyl ring.

**Table:**

*D*—H···*A*	*D*—H	H···*A*	*D*···*A*	*D*—H···*A*
N1—H1N1···O2^i^	0.86	2.12	2.969 (3)	170
C13—H13A···Cg1^ii^	0.97	2.62	3.566 (3)	166

Symmetry codes: (i) *x*, −*y*+2, *z*−1/2; (ii) *x*+1/2, *y*−1/2, *z*.
